# Beyond dose: Pulsed antibiotic treatment schedules can maintain individual benefit while reducing resistance

**DOI:** 10.1038/s41598-018-24006-w

**Published:** 2018-04-12

**Authors:** Christopher M. Baker, Matthew J. Ferrari, Katriona Shea

**Affiliations:** 1 Department of Biology, The Pennsylvania State University, University Park, Pennsylvania, United States; 2 School of Biological Sciences, The University of Queensland, St Lucia, Queensland, 4072 Australia; 3 CSIRO Ecosystem Sciences, 41 Boggo Road Dutton Park, Dutton Park, QLD 4102 Australia; 4 Center for Infectious Disease Dynamics, The Pennsylvania State University, University Park, Pennsylvania, United States

## Abstract

The emergence of treatment-resistant microbes is a key challenge for disease treatment and a leading threat to human health and wellbeing. New drugs are always in development, but microbes regularly and rapidly acquire resistance. We must consider if altering how we administer drugs at the individual level could slow development of resistance. Here we use mathematical models to show that exposing microbes to drug pulses could greatly reduce resistance without increasing individual pathogen load. Our results stem from two key factors: the presence of antibiotics creates a selection pressure for antibiotic resistant microbes, and large populations of bacteria are more likely to harbor drug resistance than small populations. Drug pulsing targets these factors simultaneously. Short duration pulses minimize the time during which there is selection for resistance, and high drug concentrations minimize pathogen abundance. Our work provides a theoretical basis for the design of *in vitro* and *in vivo* experiments to test how drug pulsing might reduce the impact of drug resistant infections.

## Introduction

By the year 2050, antibiotic resistance is expected to cause 10,000,000 deaths per year^1^, posing a major problem for disease treatment^2^ as pathogens become resistant to certain drugs^3–5^. Developing new treatments is a way forward^6–8^, but resistance can evolve quickly, sometimes even before drugs are approved^9^. In fact, it has been argued that slowing the evolution of antibiotic resistance can be more effective than developing new drugs^10^. Hence, we need to think carefully about how we administer drugs at the individual patient level, as novel treatment strategies could curb resistance evolution at the population level^11^.

The prevailing treatment strategy for bacterial infections is to administer antibiotics at high concentrations^12^. The rationale is that the ensuing high drug concentration will kill all microbes quickly, so that there is no chance for resistance to develop, while treatments at lower concentrations can assist the emergence of resistance by creating selection pressure for resistant strains^13^. Consequently, a considerable amount of experimental effort has gone into finding the drug concentrations required to suppress microbes and prevent resistance^14–17^. Unfortunately, in some circumstances it is not possible to treat at sufficiently high doses. There is a need to avoid patient side effects; the greatest drug concentration with which a patient can cope is termed the *maximum tolerable dose*. Some recent theoretical work has suggested that, in certain cases, treating at the maximum tolerable dose also leads to resistance and therefore more moderate treatment strategies could be used^18,19^. However, these strategies could lengthen patient recovery times, depending on the particular situation^20^.

Exploring additional possible management strategies is warranted. Microbes may lose fitness when acquiring *de novo* resistance, as the ability to cope with antibiotics often takes energy away from other processes^21^. Hence, in the absence of treatment, sensitive strains will tend to outcompete and regulate resistance in a population. This phenomenon occurs throughout biology, including in cancer treatment and pest control, where management strategies that exploit the competition between treatment-sensitive and treatment-resistant entities are becoming increasingly common^22–24^. It may also be possible to take advantage of this phenomenon in treating microbial infections.

In this paper, we implement mathematical models of infections to test whether there could be antibiotic management strategies that exploit the competition between sensitive and resistant strains. We focus on pulsing strategies, drawing on theory for ecological disturbances, which can be defined in terms of the frequency, intensity, and duration of the disturbance^25,26^. We conceptualise drug treatments as a type of disturbance; understanding how these different aspects of disturbance interact to affect a system is vital when developing management strategies.

## Methods

To address our question about the potential for strain competition and disturbance to interact to improve antibiotic regime outcomes, we implement three different existing differential equation models of strain competition that have previously been used to explore antimicrobial treatments regimes and the evolution of resistance^19^. We describe each of the models below; parameter values are given in the Supplementary Information S3.

The first model describes the abundance of wild, *P*_*w*_ and highly resistant, *P*_*r*_, pathogens and the immune response, *I*:

1 $$\begin{array}{rcl}\frac{d{P}_{w}}{dt} & = & ({r}_{w}(c)(1-{\mu }_{w})-{\gamma }_{w}){P}_{w}-\kappa {P}_{w}I\\ \frac{d{P}_{r}}{dt} & = & ({r}_{r}(c)-{\gamma }_{r}){P}_{r}-\kappa {P}_{r}I+{r}_{w}(c){\mu }_{w}{P}_{w}\\ \frac{dI}{dt} & = & \alpha ({P}_{w}+{P}_{r})-\delta I.\end{array}$$

We note that while we present the model as a set of differential equations, we implement a stochastic version of these equations as a continuous time Markov chain via the Gillespie algorithm^27^ (Supplementary Information S1). This algorithm produces stochastic realisations of the model, which, on average, are equal to the solution of the differential equations. While our results throughout are from the stochastic model versions, we introduce the remaining models in only their deterministic form, as is conventional^19,28,29^. There are a range of technical considerations involved with using the Gillespie algorithm, which we discuss in Supplementary Information S2.

These equations allow the wild type to mutate to be highly resistant at rate *μ*_*w*_, and both strains decay at rates *γ*_*w*_ and *γ*_*r*_ respectively. The immune system affects the strains equally, with parameter *k*, and the total pathogen load causes the immune response via *α*. The parameter *δ* is the decay of the immune response. Finally the functions *r*_*w*_(*c*), and *r*_*r*_(*c*) are treatment-dependent replication rates of the different strains, which we model using the function $\,{b}_{1}(1-\,\tanh ({b}_{2}(c-{b}_{3})))$, where *b*_1_, *b*_2_ and *b*_3_ are constants, following Day and Read^19^. This results is a time-dependent replication rate; however, as the drug concentration is piecewise constant this can be addressed in the model and we find that is has a trivial impact on simulations via the Gillespie algorithm (Supplementary Information 2). We also note that other functional forms, with different appropriate underlying biochemical bases, could instead be used to model the effect of antibiotics on pathogen growth rates^18,30^. In our simulations, we do not vary *c* continuously (it is either constant or 0), so the actual function shape does not affect the simulation. Rather, these general results require only that the different strains have different replication rates, depending on whether there is treatment or not. The model assumes that wild type pathogens have a greater replication rate when *c* = 0 than the other strains, while the highly resistant strain has the highest replication rate as *c* increases.

The second model introduces pathogens with intermediate resistance to antibiotics, *P*_*i*_, to the system of differential equations:

2 $$\begin{array}{rcl}\frac{d{P}_{w}}{dt} & = & ({r}_{w}(c)(1-{\mu }_{w})-{\gamma }_{w}){P}_{w}-\kappa {P}_{w}I\\ \frac{d{P}_{i}}{dt} & = & ({r}_{i}(c)(1-{\mu }_{i})-{\gamma }_{i}){P}_{i}-\kappa {P}_{w}I+{r}_{w}(c){\mu }_{w}{P}_{w}\\ \frac{d{P}_{r}}{dt} & = & ({r}_{r}(c)-{\gamma }_{r}){P}_{r}-\kappa {P}_{r}I+{r}_{i}(c){\mu }_{i}{P}_{i}\\ \frac{dI}{dt} & = & \alpha ({P}_{w}+{P}_{i}+{P}_{r})-\delta I.\end{array}$$

These equations have the exact same structure as Eq. (1), with the addition of parameters associated with intermediate resistance. This means that it is not possible to mutate directly from wild type to highly resistant. Rather, wild type mutates to intermediate resistance at rate *μ*_*w*_, which can subsequently mutate to highly resistant at rate *μ*_*i*_.

The third model includes resource competition. We revert to two pathogen strains, wild, *P*_*w*_, and highly resistant, *P*_*r*_. The immune system is omitted from the model, and instead a resource, *R*, is included in the system of differential equations:

3 $$\begin{array}{rcl}\frac{dR}{dt} & = & \theta -\delta R-{r}_{w}(c){P}_{w}R-{r}_{r}(c){P}_{m}R\\ \frac{d{P}_{w}}{dt} & = & {r}_{w}(c)(1-{\mu }_{w}){P}_{w}R-{d}_{w}{P}_{w}\\ \frac{d{P}_{r}}{dt} & = & {r}_{r}(c){P}_{r}R-{d}_{r}{P}_{r}+{r}_{r}(c){\mu }_{w}{P}_{w}.\end{array}$$

As with Eq. (1), the wild type is able to mutate to become resistant, and both strains have constant death rates, governed by *d*_*w*_ and *d*_*r*_. The key differences in the growth rate are the inclusion of the resource and the omission of the immune system. The growth rates *r*_*w*_(*c*) and *r*_*r*_(*c*) behave in the same way as before, but they are also scaled by the abundance of resources. The resource itself is produced at a constant rate, *θ*, and decays at rate *δR*. Any growth of *P*_*w*_ or *P*_*r*_ requires resources to be available, and this growth drains the resource.

**Figure 1:**
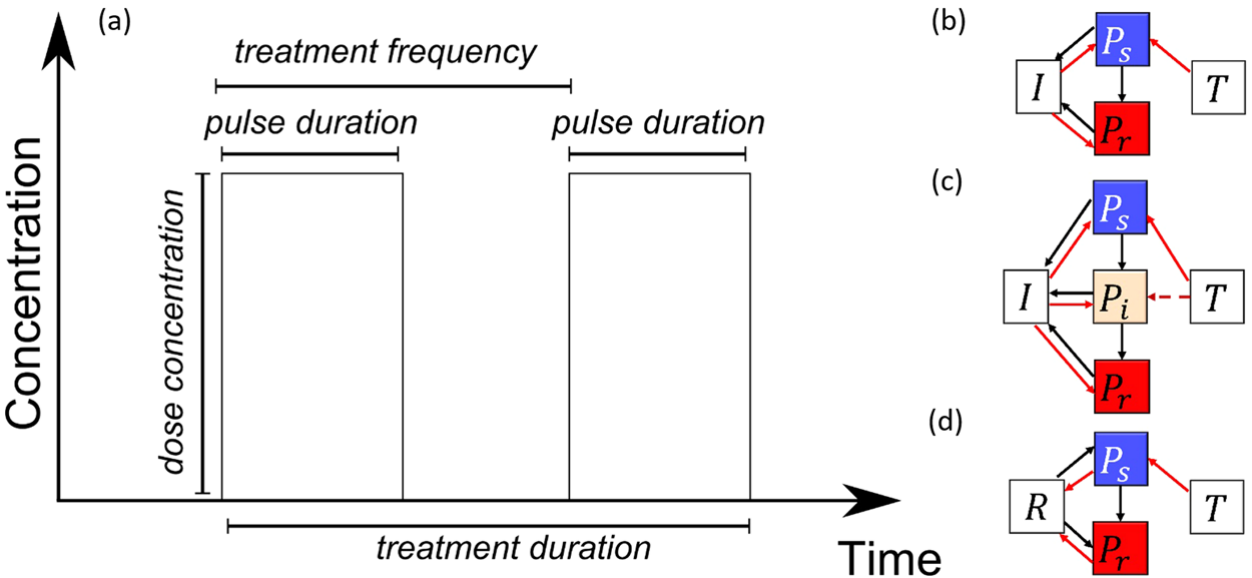
(**a**) Treatment schedules. The dose concentration is the amount of drug administered in each pulse. The treatment frequency is number of pulses per unit time, meaning the time between pulses the reciprocal of the treatment frequency. The pulse duration is how long each pulse lasts and we measure this as a fraction of the time between pulses. Finally, treatment duration is the total time window where treatment occurs. (**b**–**d**) The different model structures that we use^19^. *P*_*s*_, *P*_*i*_ and *P*_*r*_ are pathogen strains which are sensitive to treatment, have intermediate resistance, and are highly resistant to treatment, respectively. *T* is the treatment being applied, *I* is the immune response and *R* is resources. In each case, sensitive microbes can mutate to become more resistant to treatment. In (**b**) and (**c**), the immune system suppresses all strain types. In (**d**), the strains compete for resources.

System perturbations can generally be described in terms of their frequency (e.g. how often the antibiotic is administered), intensity (e.g. how much antibiotic is dispensed), timing (e.g. when the antibiotic is applied), duration (e.g. for how long the antibiotic treatment lasts). Each of these can have different effects on the system dynamics, mediated by their interactions with other aspects. In the context of antibiotics, we here vary four aspects of the treatment regime: the dose concentration; the pulse frequency; the pulse duration; and the total treatment duration (Fig. 1a). We define dose in terms of its impact on the pathogen population growth rate in all the models; hence, it is dimensionless in this representation. We define the pulse duration to be the fraction of time between the start of pulses, such that a pulse duration of 1 corresponds to constant treatment, while a pulse duration of 0.5 means that treatment occurs 50% of the time. We vary each of these aspects to test how they affect the total pathogen burden (the pathogen abundance, integrated across the treatment window) and the probability of resistance emerging (defined as *P*_*r*_ > 100 for models one and two, and *P*_*r*_ > 300 for the resource competition model, following^19^). For each case, treatment begins once the total pathogen abundance reaches 100 (${P}_{w}+{P}_{i}+{P}_{r}\ge 100$), and we run the model up to *T* = 100.

### Data availability

All MATLAB code is publically available https://doi.org/10.6084/m9.figshare.4287809.v1. There was no data used in this work.

## Results

**Figure 2:**
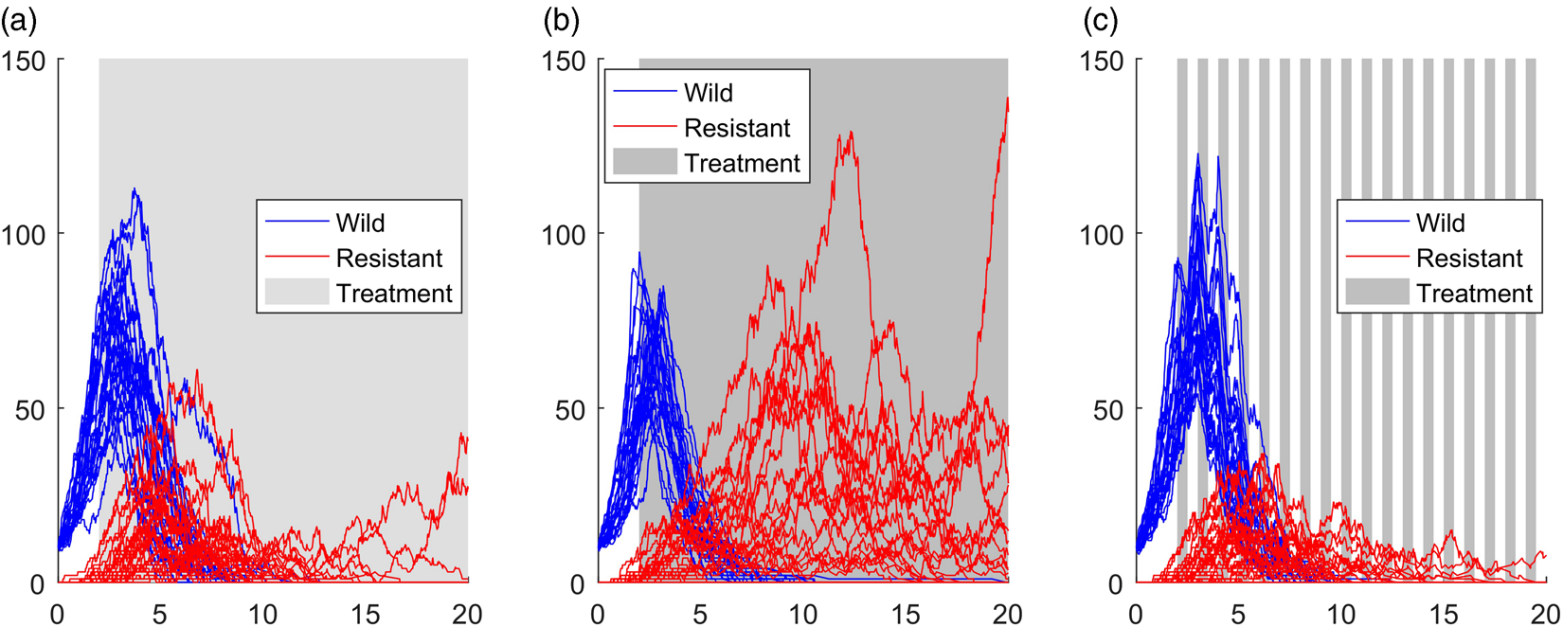
Individual realisations from the model with a wild strain (blue), an intermediate resistant strain (not shown), a highly resistant strain (red) and immune response (not shown). (**a**) and (**b**) have constant treatment with dose = 0.3 and dose = 0.5 respectively. (**c**) Has a pulse duration of 0.5 and dose = 0.5. The pulsed treatment has less resistance, but the abundance of wild type is higher than in the constant treatments.

In this section we focus on results from model two (Fig. 1c; three-strain plus immune system); results from the other two models are very similar and are presented in the Supplementary Information S4. Figure 2 shows example simulations from the three-strain model (Fig. 1c). A constant treatment using a high dose results in relatively low wild type (sensitive) abundances, but does result in much higher abundances of the resistant strain, compared to a constant treatment of a low dose. Figure 2c shows the outcome when we shift to a pulsed strategy; the abundance of the resistant strain is reduced, compared to both constant treatment strategies.

**Figure 3:**
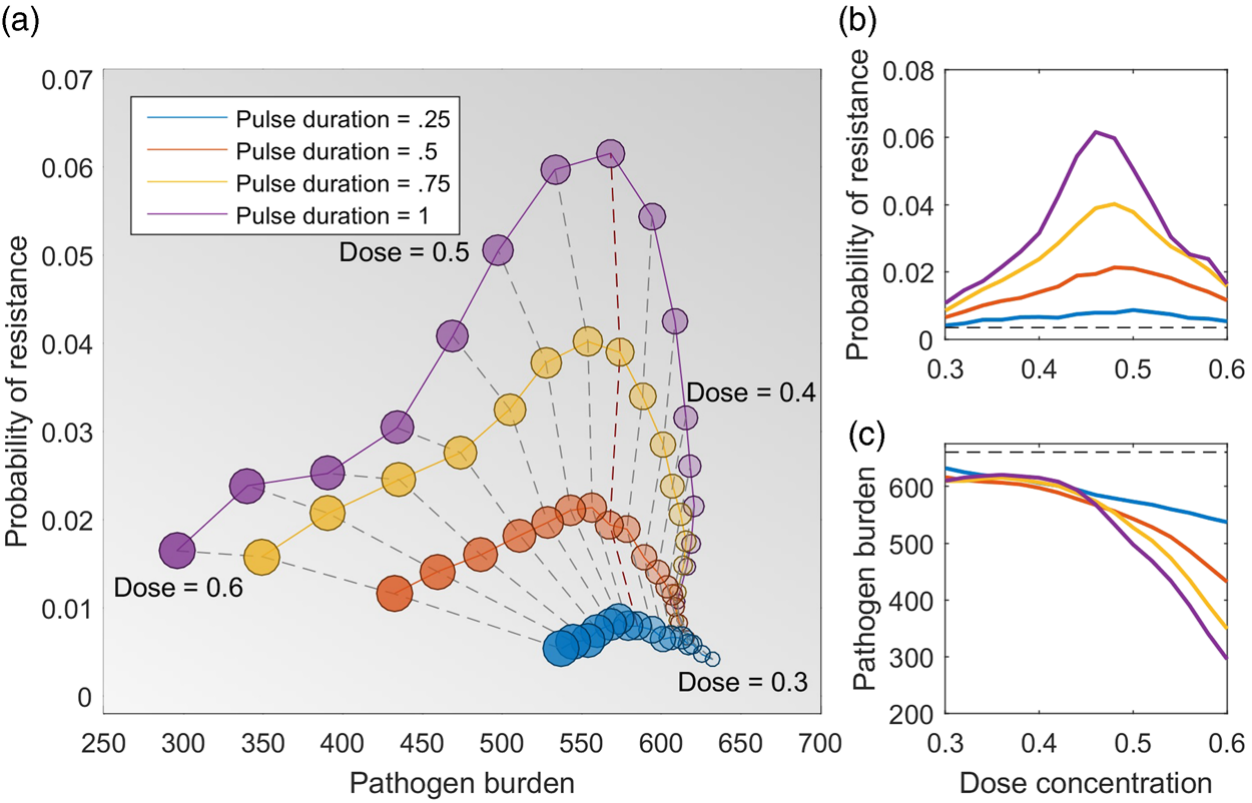
The probability of resistance and the total pathogen burden for different treatment strategies using the three-strain model (Fig. 1c). Here the total treatment window is 20 days and the treatment frequency is 1. (**a**) Each circle is the average of 100,000 simulations, and the dashed lines connect treatments that have the same dose concentration. The ideal treatment strategies are in the bottom left of the figure, as these minimise the probability of resistance and the pathogen burden. However, these strategies require very high doses and are not always possible due to drug toxicity. The red dashed line at dose = 0.46 marks where altering the pulse duration (at or above 0.5) does not affect the pathogen burden, but does change the probability of resistance. At higher doses, there is a trade-off between lowering the pathogen burden and lowering the probability of resistance. (**b**,**c**) Show the probability of resistance and pathogen burden separately, both as a function of the dose concentration, and the black line in (**c**) indicates a region where all strategies are almost equivalent in pathogen burden, despite very different probabilities of resistance.

We ran simulations across a wide range of dose concentration, pulse duration and pulse frequencies. For each combination, we calculate the probability of resistance emerging and the total pathogen burden. In Fig. 3, we show results for model two; again the results for the other two models are similar and are given in the Supplementary Information S4. We find that very high doses result in low pathogen burdens and low probability of resistance, but this regime often requires doses above the maximum tolerable dose. The pulsed treatments lower the probability of resistance compared to the constant treatment, particularly for moderate dose concentrations. Again, we find the same qualitative behaviour with each of the models; reducing pulse duration reduces the probability of resistance (see Supplementary Information S4). Importantly, there appears to be a dose concentration threshold: at doses below the threshold, pulsed treatment reduces the probability of resistance without adversely affecting the pathogen burden. For example, if the maximum dose that a patient can tolerate is 0.46, then the probability of resistance can be reduced from 0.06 to 0.01 by shifting from a continuous dose to a pulse duration of 0.5, without affecting the pathogen burden (Fig. 3, red line). However, reducing the duration further would incur an increased patient bacterial burden.

Varying the dose duration without changing the treatment duration means that the total amount of drug used varies between simulations. Hence, we also ran simulations where we used a constant number of doses with a constant dose duration, but varied the treatment frequency, so that the total drug use was kept constant (see Supplementary Information S4). We obtained very similar results for these simulations. The probability of resistance emergence peaks at moderate dose. Interestingly, however, in these total drug-controlled simulations, the total pathogen burden is generally lower for the pulsed strategies compared to the constant strategy.

## Discussion

In this paper we used existing stochastic models of bacterial dynamics and resistance evolution^19^ to test whether drug pulses could be a viable treatment option. We analysed three different model structures, which include immune response, resource competition, and multiple levels of resistance (Fig. 1b,c and d; Supplementary Information S4). Our aim was to find treatment regimes that reduce the emergence of resistance without increasing an individual patient’s total pathogen burden. We found that there are indeed a range of dose concentrations where moving to a pulsed strategy decreased resistance, without greatly increasing pathogen burden. However, above a dose threshold we find a trade-off between constant and pulsing treatments, where a constant dose results in a lower total pathogen burden, but a high probability of resistance compared to pulsing. A clear question that follows is whether these results require a specific pulse duration or not. We find that our results (Fig. 3) are driven by the proportion of time that treatment is applied, provided that the drug pulse is reasonably short (see Supplementary Information S4). For example, the outcomes for a treatment frequency of once per day are the same as treatment twice per day with a shorter dose duration. While this is true across the range of treatment frequencies that we considered (from four times per day to every second day), this would not hold for very long dose durations, as these would begin to resemble constant treatments.

Our parameter values do not come from empirical studies, but from previous theoretical work^19^. However, we wish to emphasise that our assumptions in parameter choices are fairly conservative. First, we assume that the replication rate for wild type pathogens is higher than resistant, but the parameter value is only 1% higher, which is conservative. The second key assumption is that antibiotics affect wild type microbes at a lower concentration than resistant – which is, of course, the definition of resistance. Further, there is a wealth of empirical results that supports the qualitative features of our results (Fig. 3), the ‘inverted U’ shape, where the probability of resistance peaks at intermediate doses^19,31^. Hence, we believe that our results would hold for any system, provided that there is a cost of resistance.

These outcomes can be understood in terms of absolute and relative fitness of microbes^32^. Resistant strains have a selection advantage over wild types whenever treatment reduces the fitness of the wild type below that of the resistant strain, and when the dose concentration is below the mutant resistant concentration (i.e. the required dose concentration to suppress resistant strains). If it is not possible to force the absolute fitness of the resistant strain to be negative, then the only way to control it is to ensure that, at times, its relative fitness is less than the sensitive strain. It is important to note that missed treatments are not equivalent to these pulsed strategies and it has been established that missing doses can lead to resistance in long-term treatments, such as for HIV^33^. As these treatments are sustained for long periods without resistance emerging, it is likely that the dose concentrations are well above the mutant resistant threshold; in such circumstances, pulsing would not help.

There is a foundation for testing these aspects of treatment experimentally. For example, it has been found experimentally that reducing the total duration of treatment can reduce the prevalence of resistance without impacting the disease burden^11^, and experiments have found that using multiple drugs to treat disease can be effective^34,35^. However, pulsed treatments have yet to be deliberately and systematically tested empirically. For systems with multiple drugs, the number of potential single and combination strategies increases quickly, and modelling has an important role to play in indicating which type of strategies will be likely to succeed. Similar ideas are also being explored in cancer treatment, where high doses of chemotherapy hasten the evolution of resistance to treatment^36^, and astute use of multiple drugs seems to be a way forward^37,38^. However, it has also been found experimentally that altering treatment doses, or even skipping doses, can improve individual outcomes substantially^23^. The success of these novel treatment strategies in cancer treatment suggests that we may make similar progress using novel antibiotic treatment schedules.

The drug strategies that we suggest here have pharmacokinetics and pharmacodynamics that are idealized representations of those usually observed. However, the absorbance and excretion rates of some drugs may be a reasonable approximation to our pulses. The type of drug that would best fit with our model would have a short half-life and might be too toxic to use continuously above the mutant resistant concentration; two features that would generally be avoided in drug development, but that might be viable for pulsed treatment regimes. It may also be possible to use combinations of drugs that interact to produce suitable drug pulses. Future theoretical explorations should also be extended to more realistic dose and drug dynamics.

Antibiotic resistance poses one of the greatest risks to human health and well-being. It is unlikely that there will be a single solution to the problem, and continuing drug development, limiting agricultural use of antibiotics and prudent use of antibiotics in human health are all important measures. There is no strong precedent for studying pulsed treatments in drug studies, however, there is a robust history of research on perturbations in ecology^25,39^; this could be used as a basis for novel experimental design for *in vitro* and *in vivo* experiments. While pulsed treatments will not succeed in every situation, our work suggests that, in some cases, they may be able to reduce antibiotic resistance without any negative impact on the patient. This is a shift from previous thinking, where the aim of any treatment strategy is to avoid doses below the mutant resistant threshold^13,19^. Our approach thus opens the way to additional testing of drugs currently deemed unacceptable, and to reusing old or existing drugs in new ways. Exploring these types of novel treatment regimes may thus usefully contribute to the fight against antibiotic resistance.

## Electronic supplementary material

Supplementary material
