# Supplementary material for: Beyond dose: Pulsed antibiotic treatment schedules can maintain individual benefit while reducing resistance
Source: Sci Rep. 2018 Apr 12;8:5866. doi: 10.1038/s41598-018-24006-w (PMC5897575; doi:10.1038/s41598-018-24006-w)
Supplement: Supplementary file 1 — Supplementary material [file 41598_2018_24006_MOESM1_ESM.docx]

**Supplementary material for “Beyond dose: Pulsed antibiotic treatment schedules can maintain individual benefit while reducing resistance”**

**Authors:** Christopher M. Baker, ­ Matthew J. Ferrari and Katriona Shea

**S1: Markov chain representation**


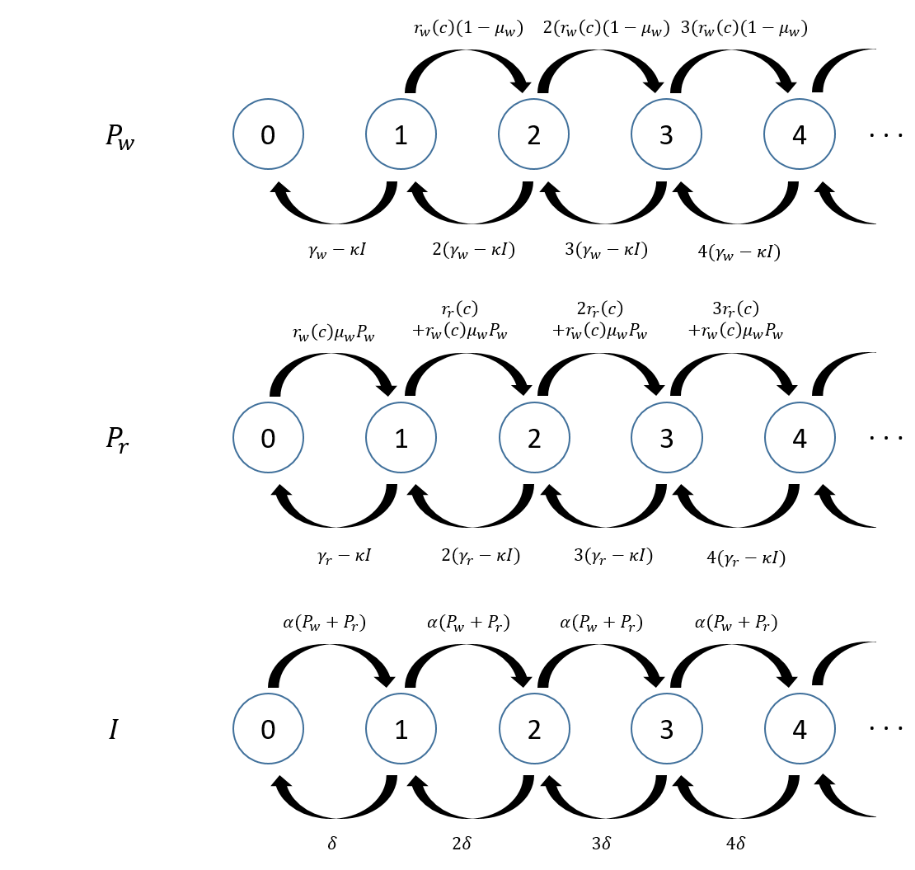


Markov chain corresponding to Eq. 1. Each row corresponds to the state variables as defined in the main text: $P_{w}$, $P_{r}$ and $I$. Each arrow represents a transition from one state to another, and the expression is the rate of that transition. The state $P_{w}=0$ is an absorbing state; once it reaches zero there is no way to increase again. This is not the case for the resistant strain, which can move from 0 to 1 via mutation from the wild population.

**S2: Gillespie implementation**

An important consideration when implementing the Gillespie algorithm is whether it is appropriate for the given equations. In our case, the models contain linear and mass action terms, which are the exact type of form for which algorithm was designed (Gillespie 1977). However, our equations also contain a time-varying term, $r(c)$. When using the Gillespie algorithm, all the rate terms are constant within any time-step, so if any rate in the true equation changes within a Gillespie time-step, there will be an associated error. In our case, however, $r(c)$ is piecewise constant, which means that there are relatively few times when the rates change. Hence, provided that our Gillespie time-steps are much shorter than the pulsing period, the associated error will be very small. The shortest pulse duration that we use is $t=0.25$, and the typical Gillespie time-step is around $t=0.0015$ – which is two orders of magnitude smaller. This means there should be no, or only trivial, issues caused by this approximation. Nevertheless, we test the potential impact of the approximation by comparing our results to an alternative algorithm. The alternate algorithm ensures that the rate parameter and the clock are both updated when the concentration changes. We do this by updating the concentration in the time-step before a change in the dose concentration, rather than updating it once the concentration has changed. Comparisons between these two algorithms for our results are given in the figures below. We tested the algorithms for dose durations of 0.25 and 0.75, dose frequencies of 0.5 and 4 and treatment durations of 20 and 50. For all these simulations, we see extremely similar results, and all simulations agree with our overall results. Hence, we conclude that approximations of our treatment of the time-varying terms are not driving any of the results in this paper.


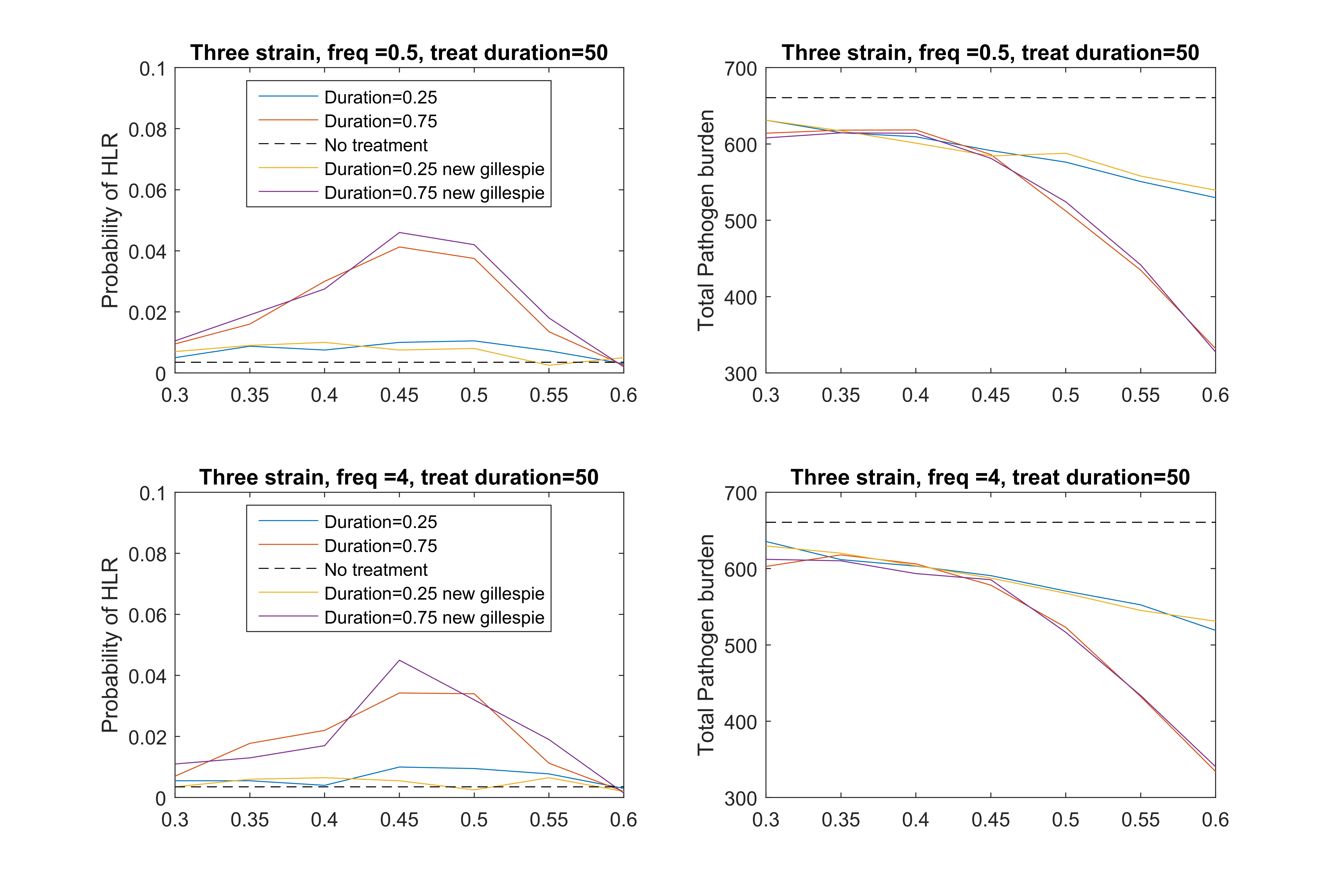

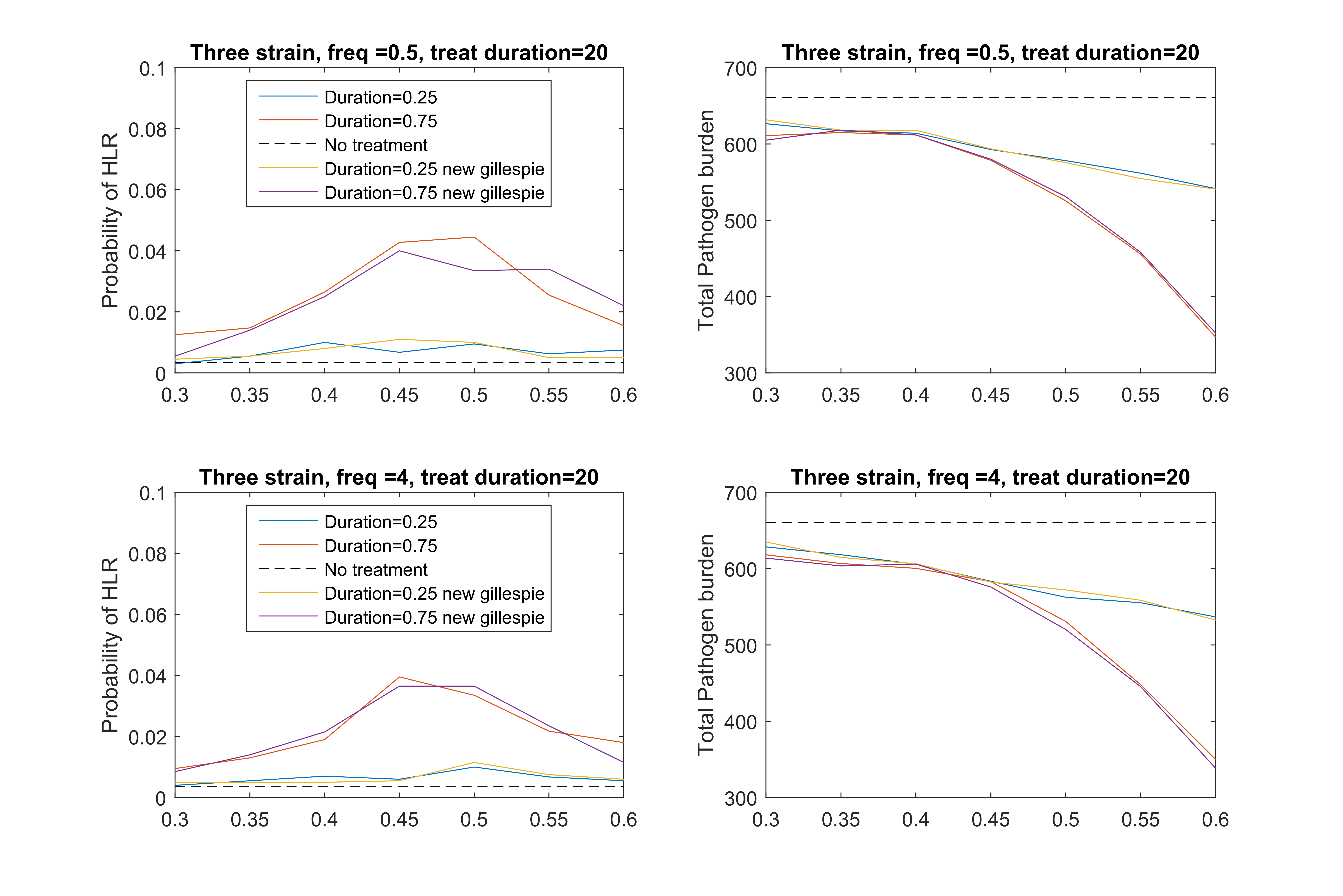


**S3: Model parameters**

**Table 1:** Parameter values for model 1.

| *Initial conditions* | |
| --- | --- |
| $P_{w}(0)$ | $10$ |
| $P_{r}(0)$ | $0$ |
| $I(0)$ | $2$ |
| *Parameters* | |
| $\mu_{w}$ | $0.01$ |
| $\gamma_{w}$ | $0.01$ |
| $\gamma_{r}$ | $0.01$ |
| $\kappa$ | $0.075$ |
| $\alpha$ | $0.05$ |
| $\delta$ | $0.05$ |
| $r_{w}\left( c \right)$ | $0.6\left( 1-\tanh\left( 15(c-0.3) \right) \right)$ |
| $r_{r}(c)$ | $0.59\left( 1-\tanh\left( 15(c-0.6) \right) \right)$ |

**Table 2:** Parameter values for model 2.

| *Initial conditions* | |
| --- | --- |
| $P_{w}(0)$ | $10$ |
| $P_{i}(0)$ | $0$ |
| $P_{r}(0)$ | $0$ |
| $I(0)$ | $2$ |
| *Parameters* | |
| $\mu_{w}$ | $0.1$ |
| $\mu_{i}$ | $0.1$ |
| $\gamma_{w}$ | $0.01$ |
| $\gamma_{i}$ | $0.01$ |
| $\gamma_{r}$ | $0.01$ |
| $\kappa$ | $0.075$ |
| $\alpha$ | $0.05$ |
| $\delta$ | $0.05$ |
| $r_{w}\left( c \right)$ | $0.6\left( 1-\tanh\left( 15(c-0.3) \right) \right)$ |
| $r_{i}(c)$ | $0.595\left( 1-\tanh\left( 15(c-0.45) \right) \right)$ |
| $r_{r}(c)$ | $0.59\left( 1-\tanh\left( 15(c-0.6) \right) \right)$ |

**Table 3:** Parameter values for model 3.

| *Initial conditions* | |
| --- | --- |
| $R(0)$ | $2000$ |
| $P_{w}(0)$ | $2$ |
| $P_{r}(0)$ | $0$ |
| *Parameters* | |
| $\mu_{w}$ | $0.01$ |
| $d_{w}$ | $2$ |
| $d_{r}$ | $2.7$ |
| $\theta$ | $200$ |
| $\delta$ | $0.1$ |
| $r_{w}\left( c \right)$ | $0.00255\left( 1-\tanh\left( 15(c-0.3) \right) \right)$ |
| $r_{r}(c)$ | $0.0025\left( 1-\tanh\left( 15(c-0.45) \right) \right)$ |

**S4: Supplementary figures**

We repeat the simulations used to produce Figure 3 in the main text with all three models and using a wider range of treatment schedules. We provide the results for all of these in this section. All of the simulations have 2000 repeats, so the lines are noisier than the results in the main text. However, it is clear that the qualitative shapes of the outcomes are consistent across models and treatments. In each case the duration is the length of the pulse duration, relative to the time between treatments. Only in the model 3 does the frequency of treatment start to affect the outcomes. However, we still see the same pattern in each of the plots.

*Model 1: two strain with immune response*


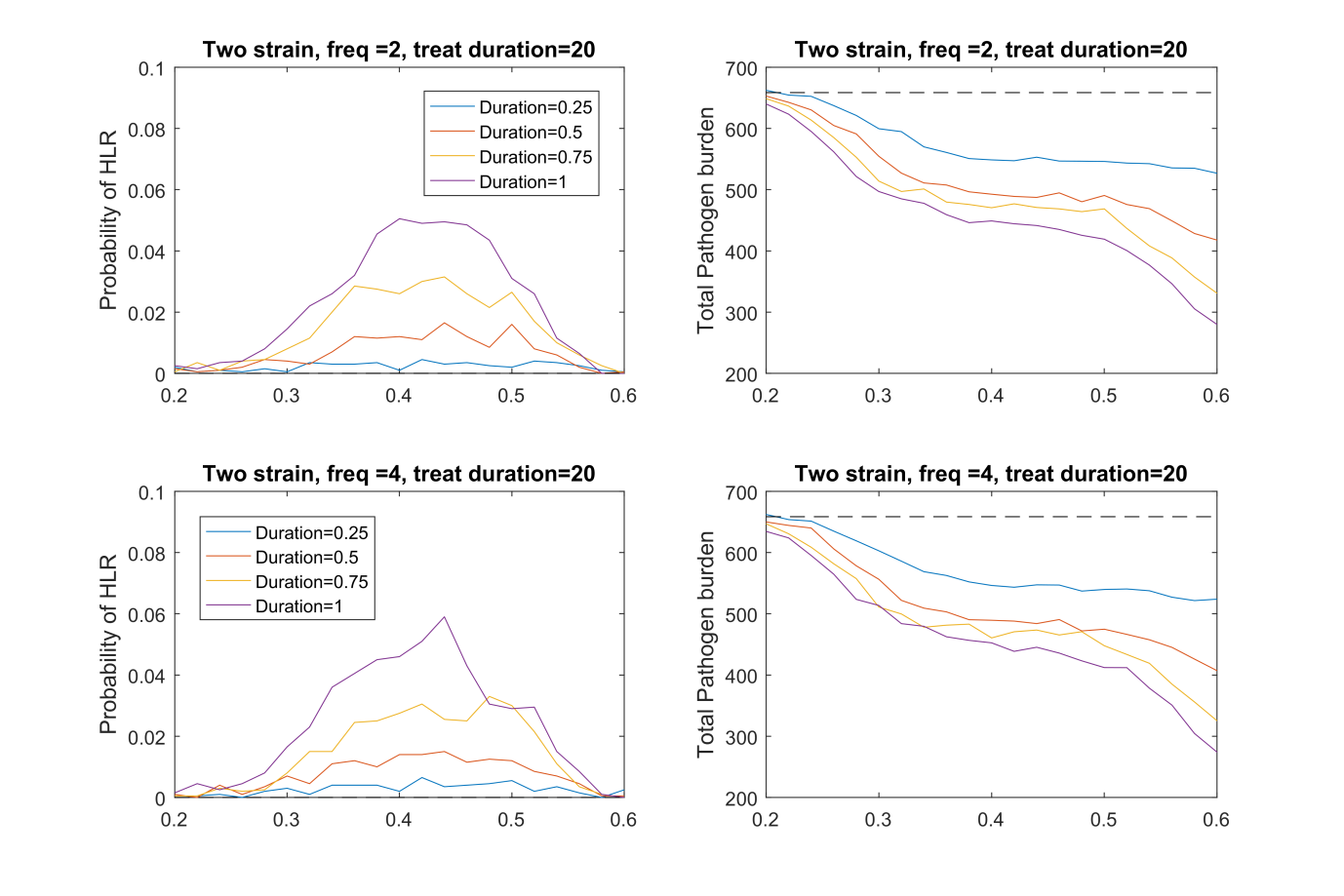

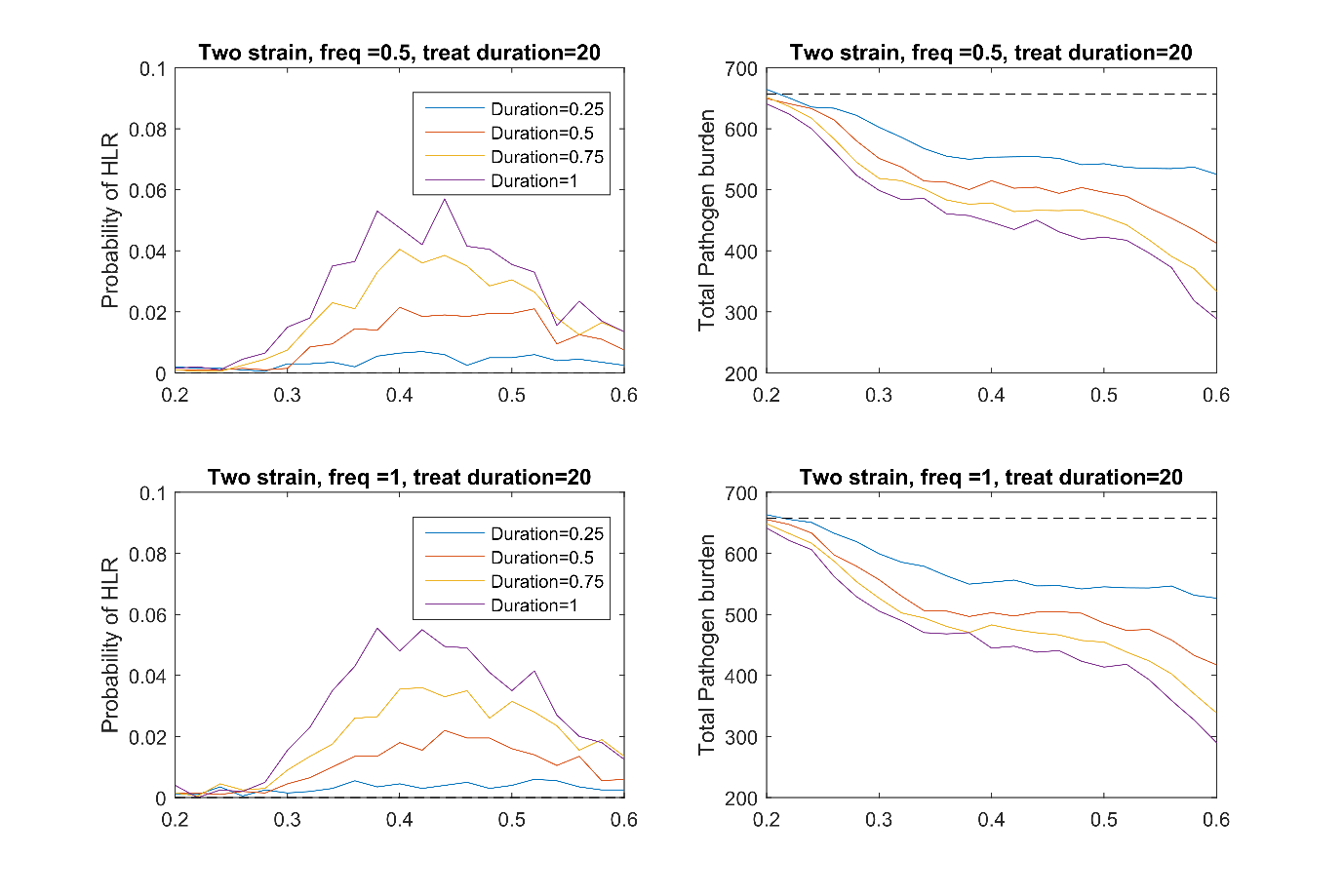

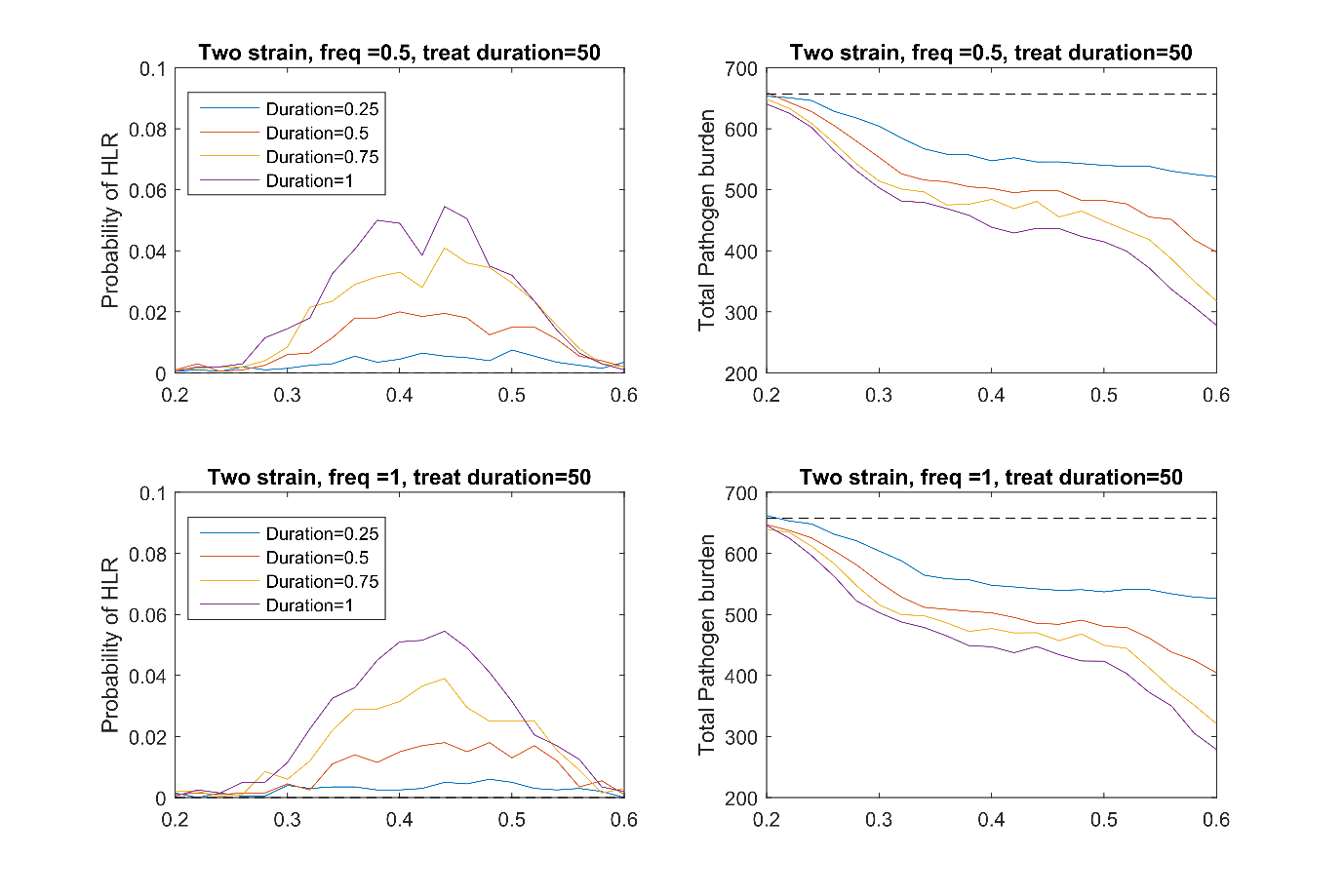

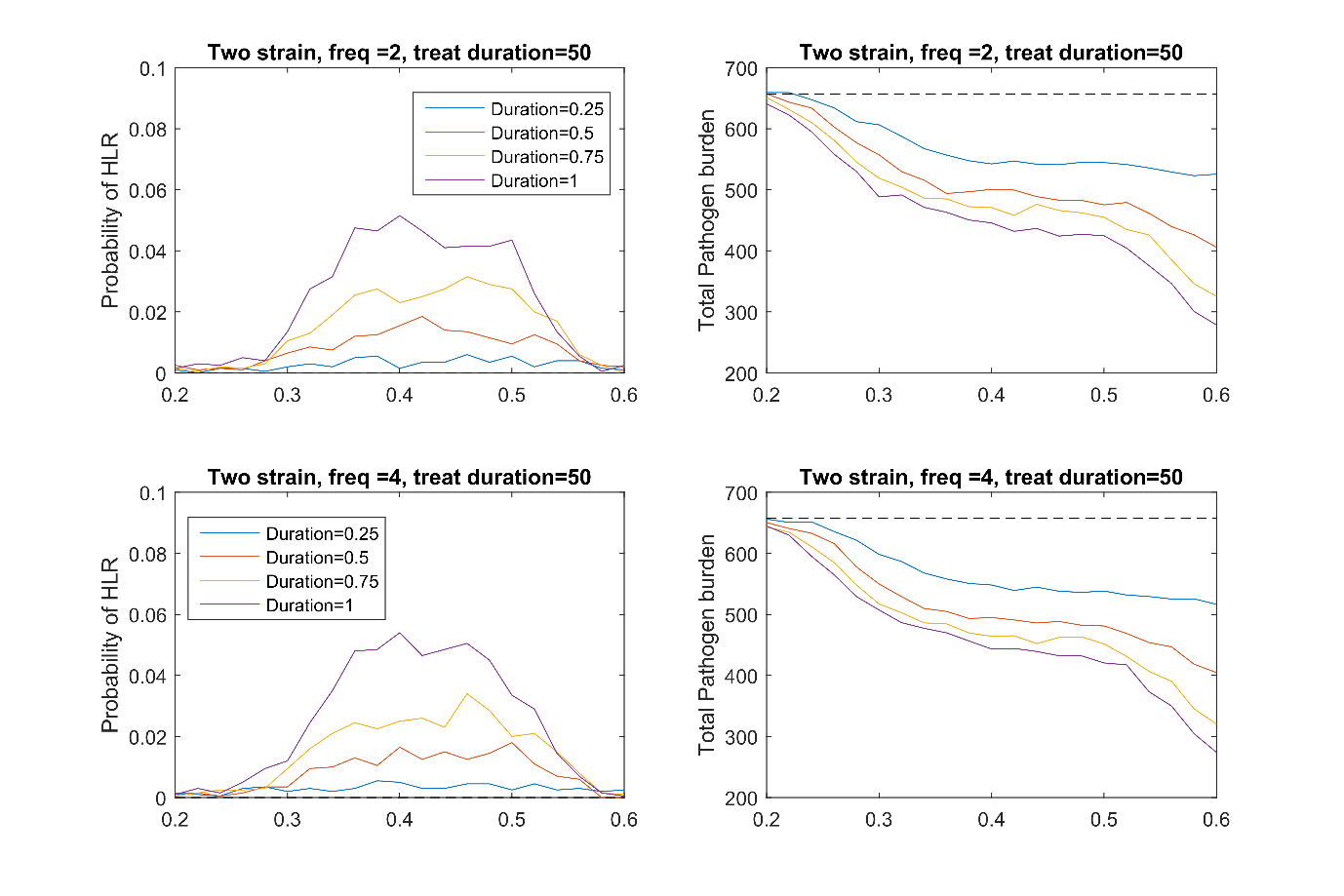

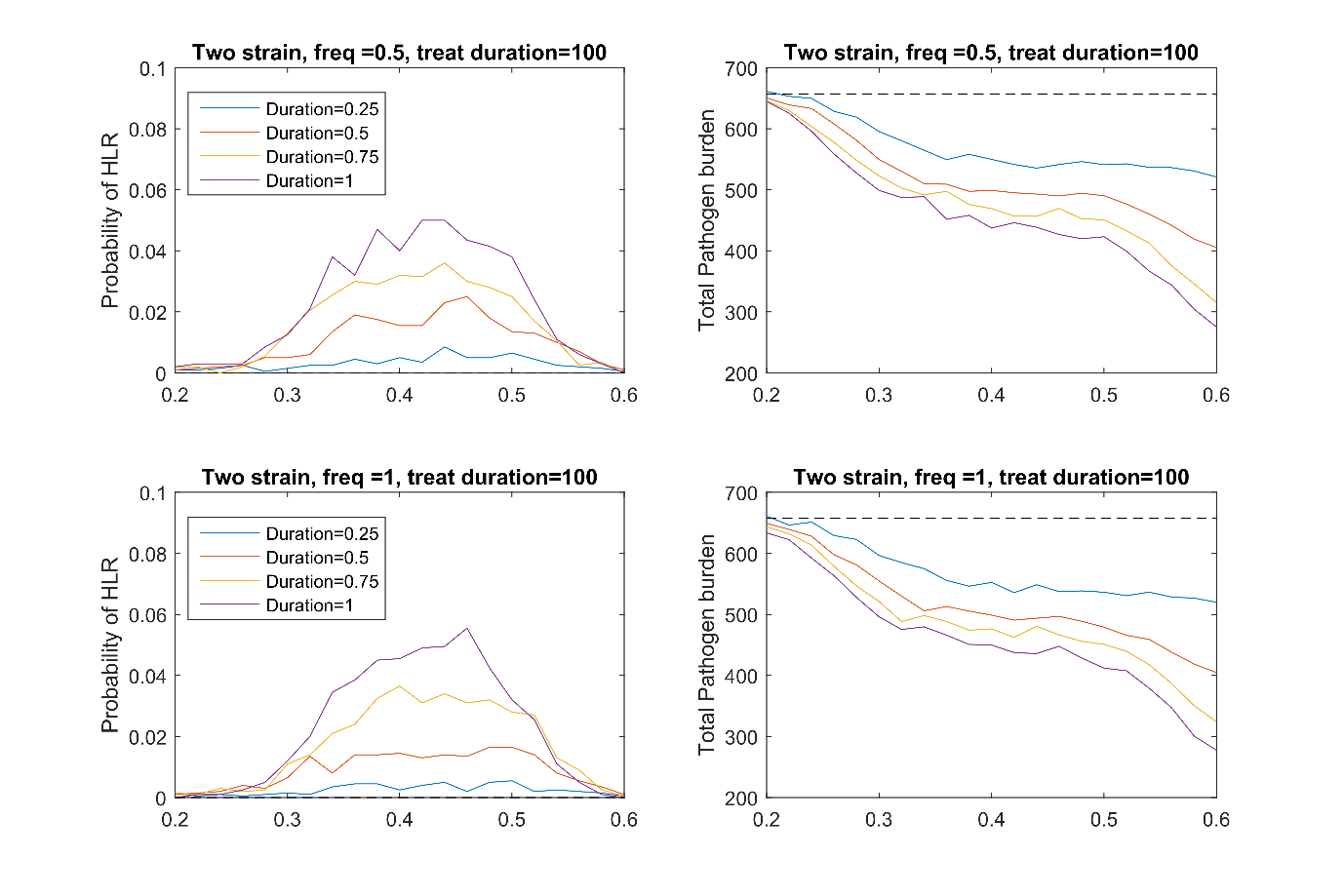

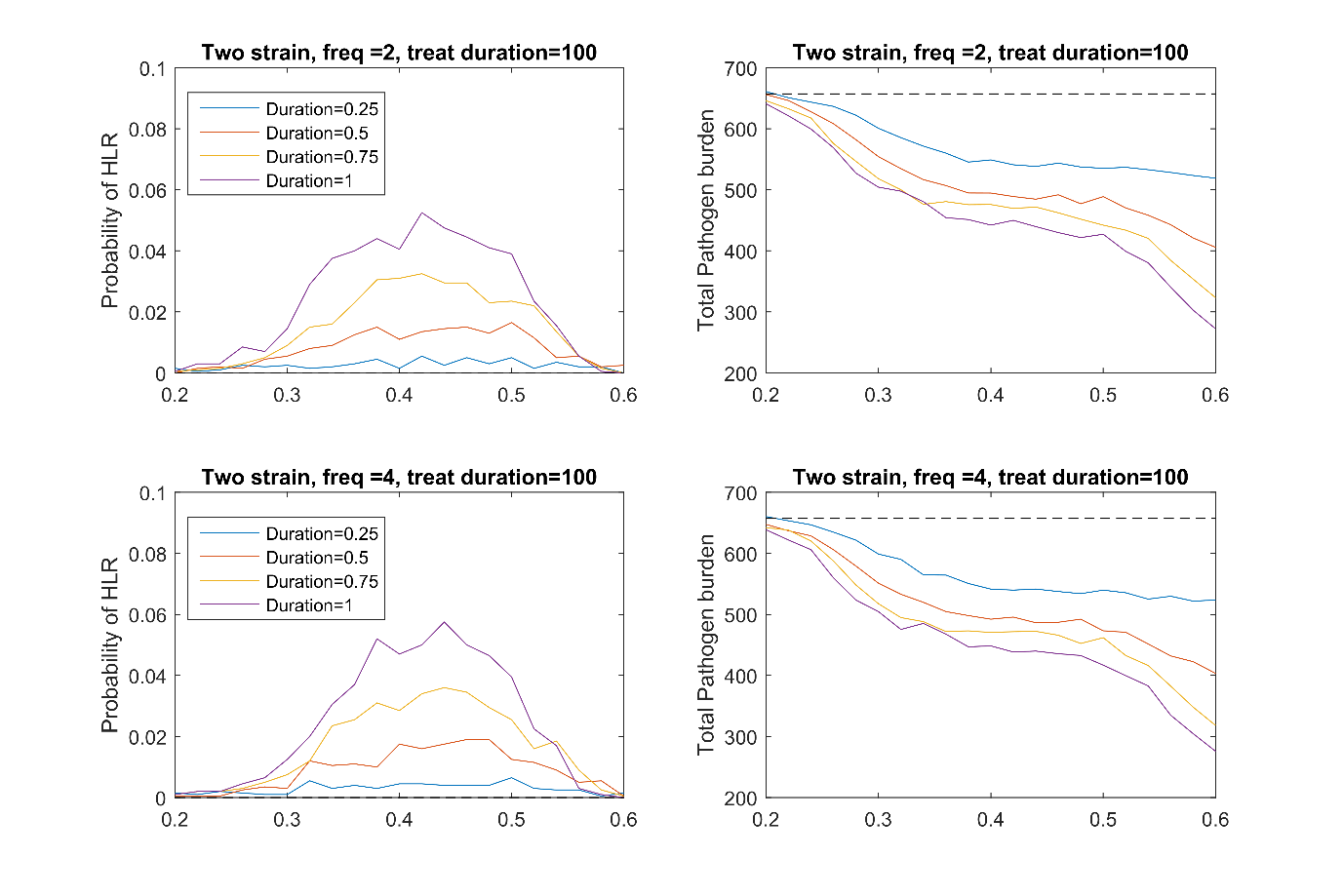
*Model 2: three strain with immune response*

*
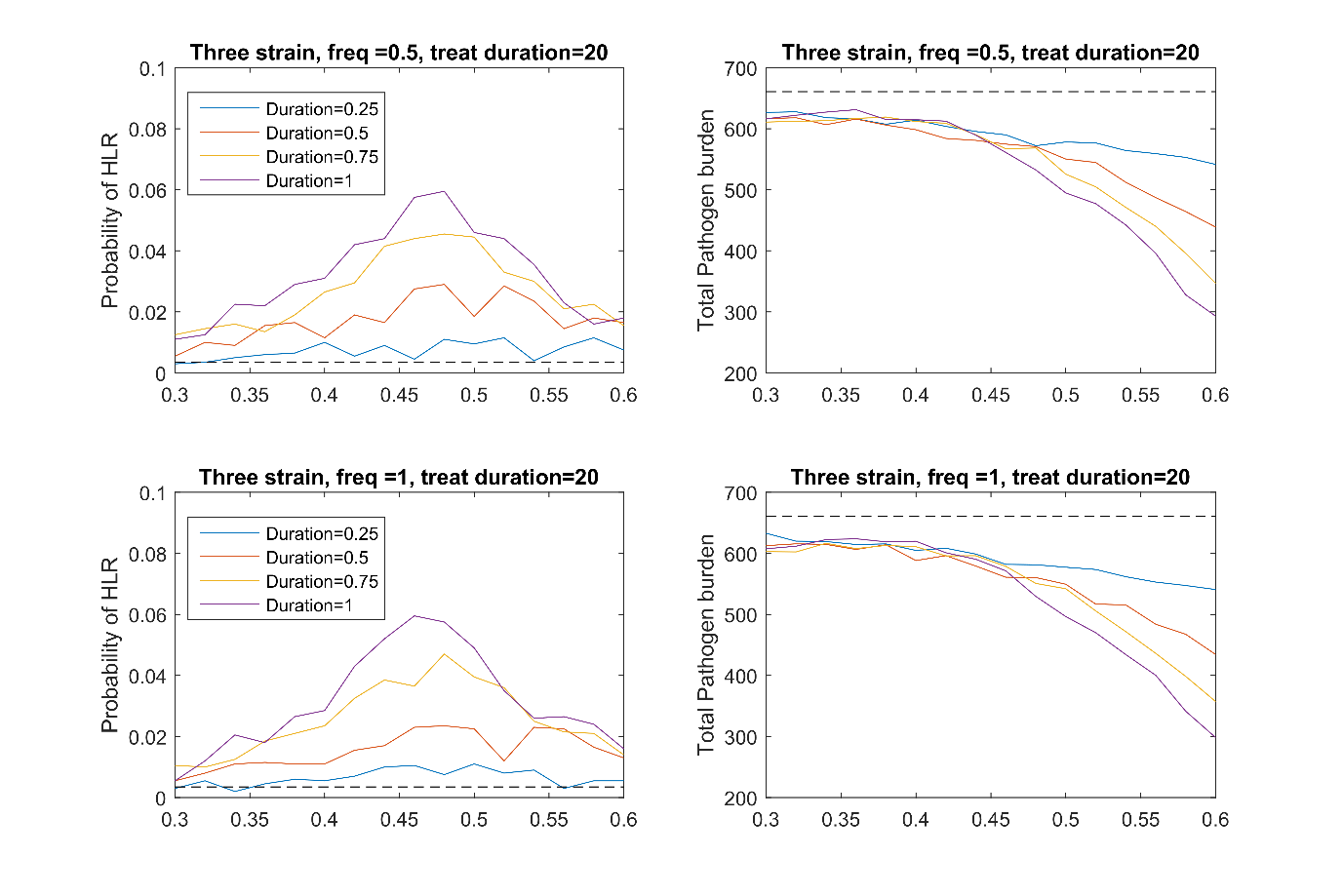

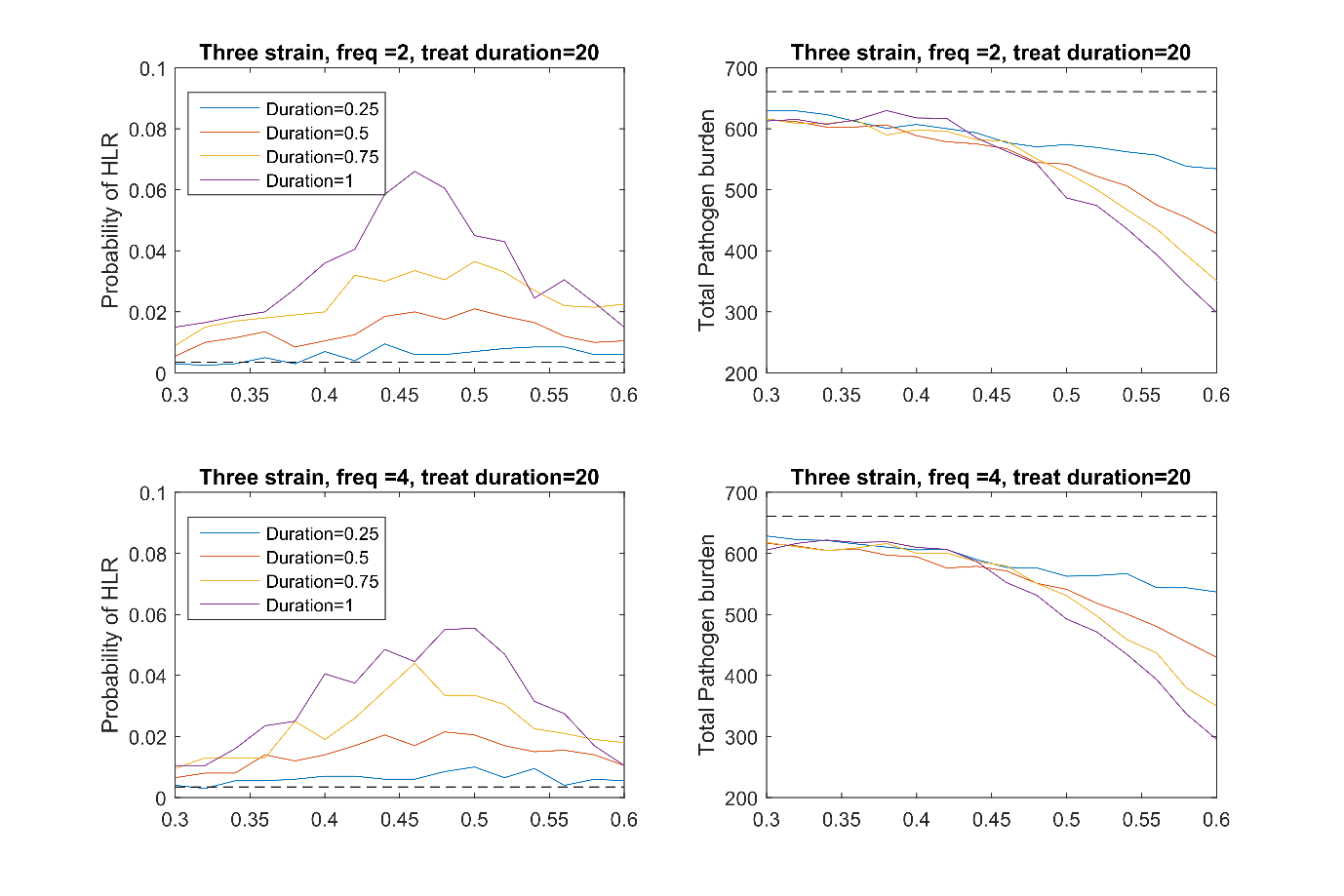

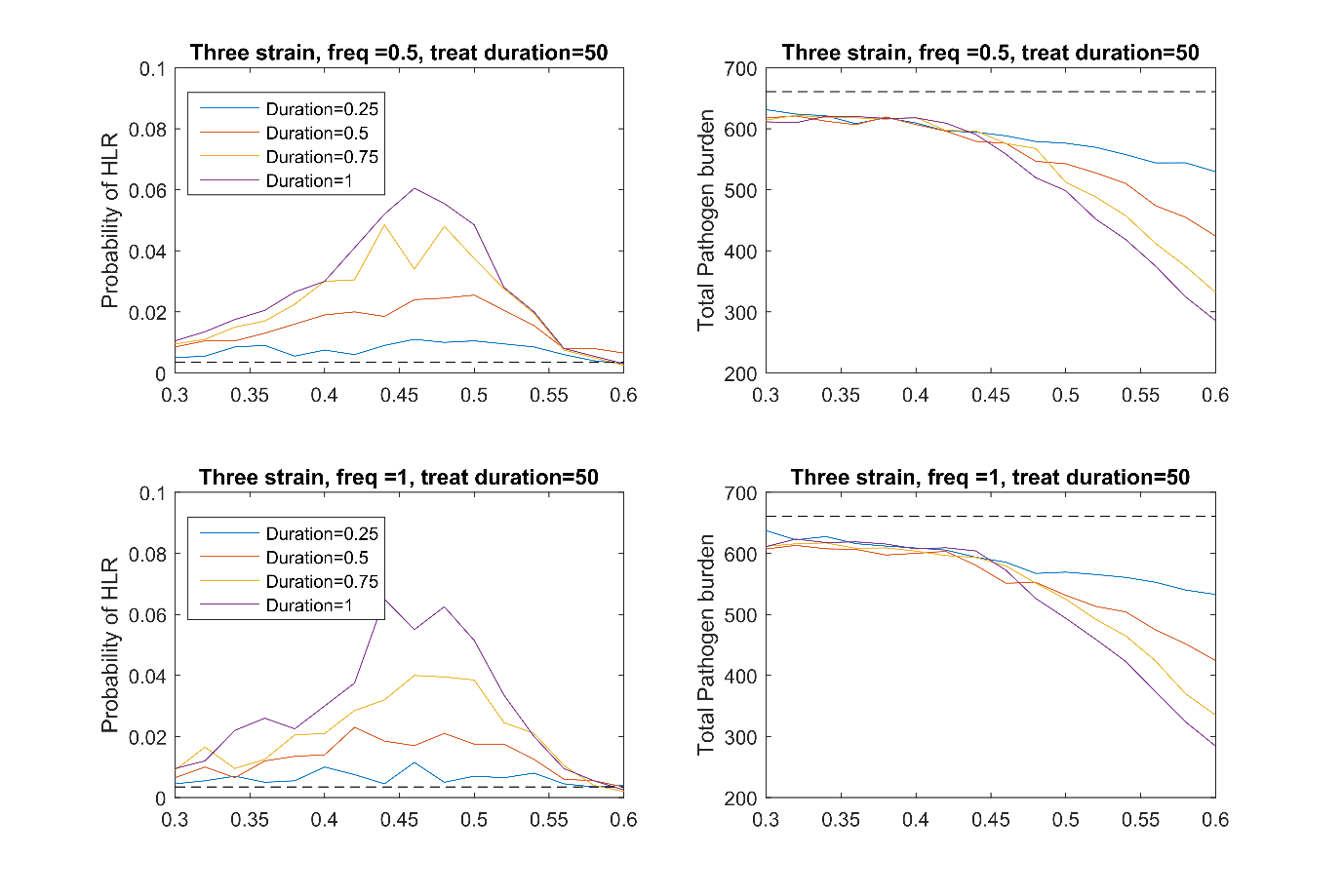

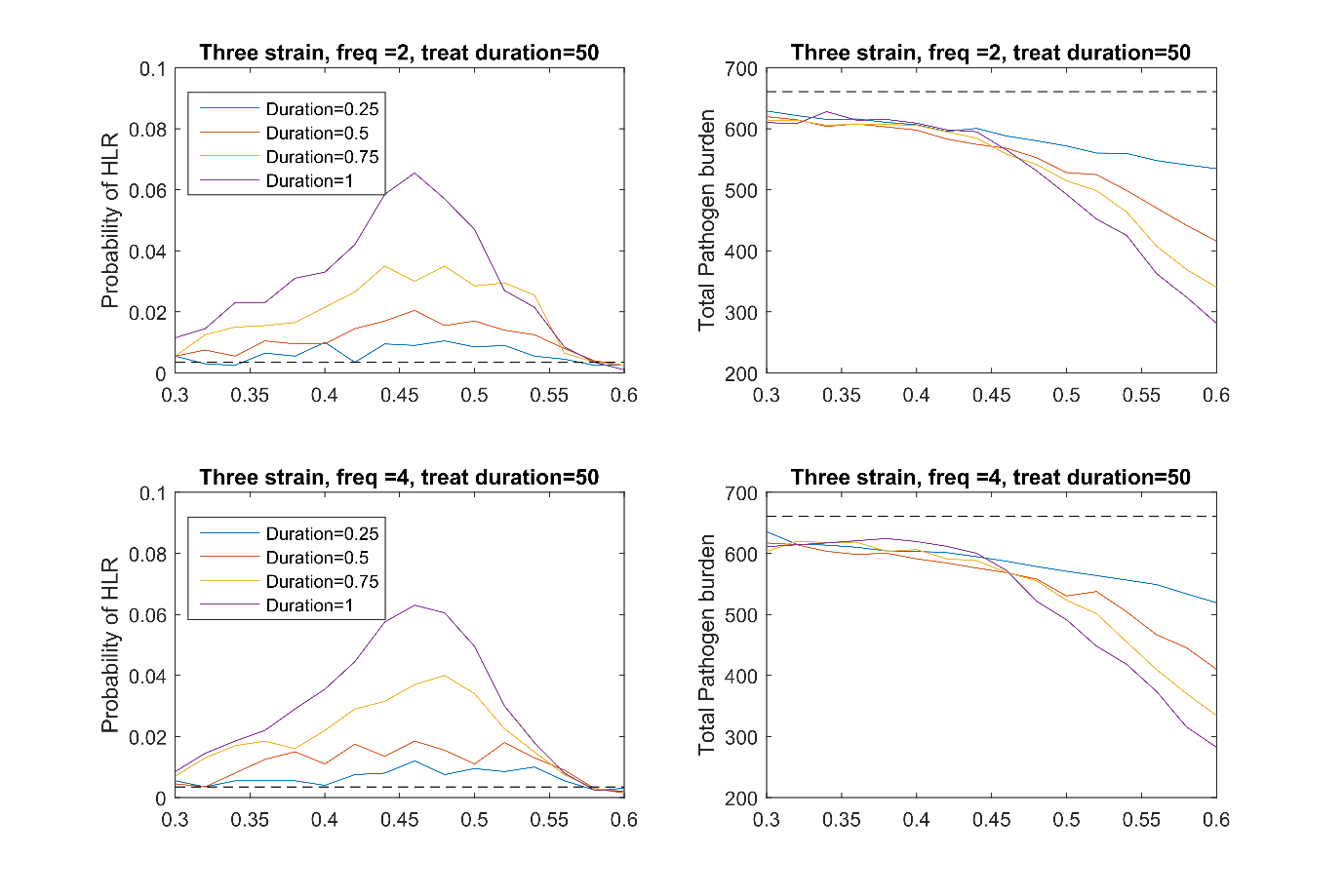

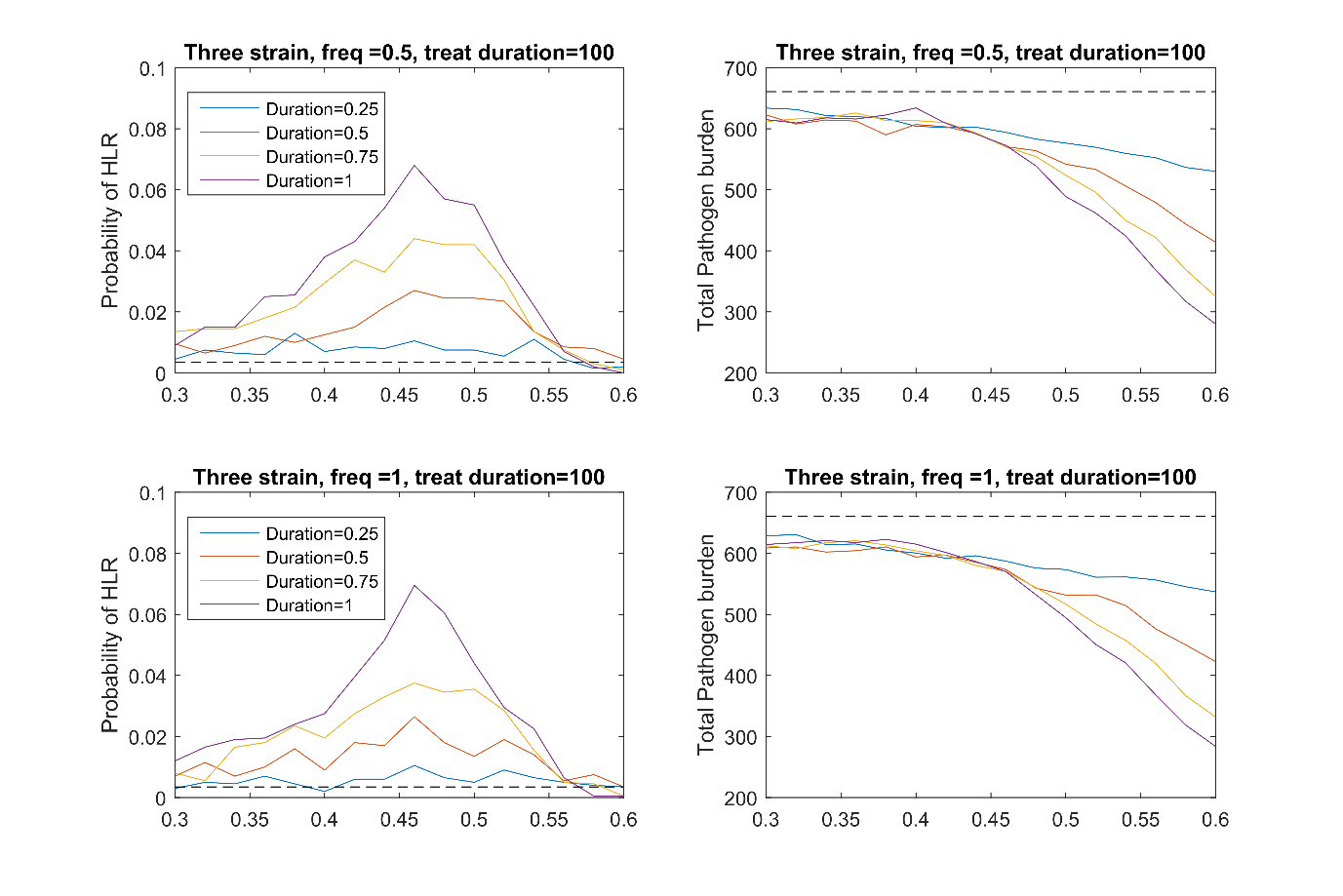

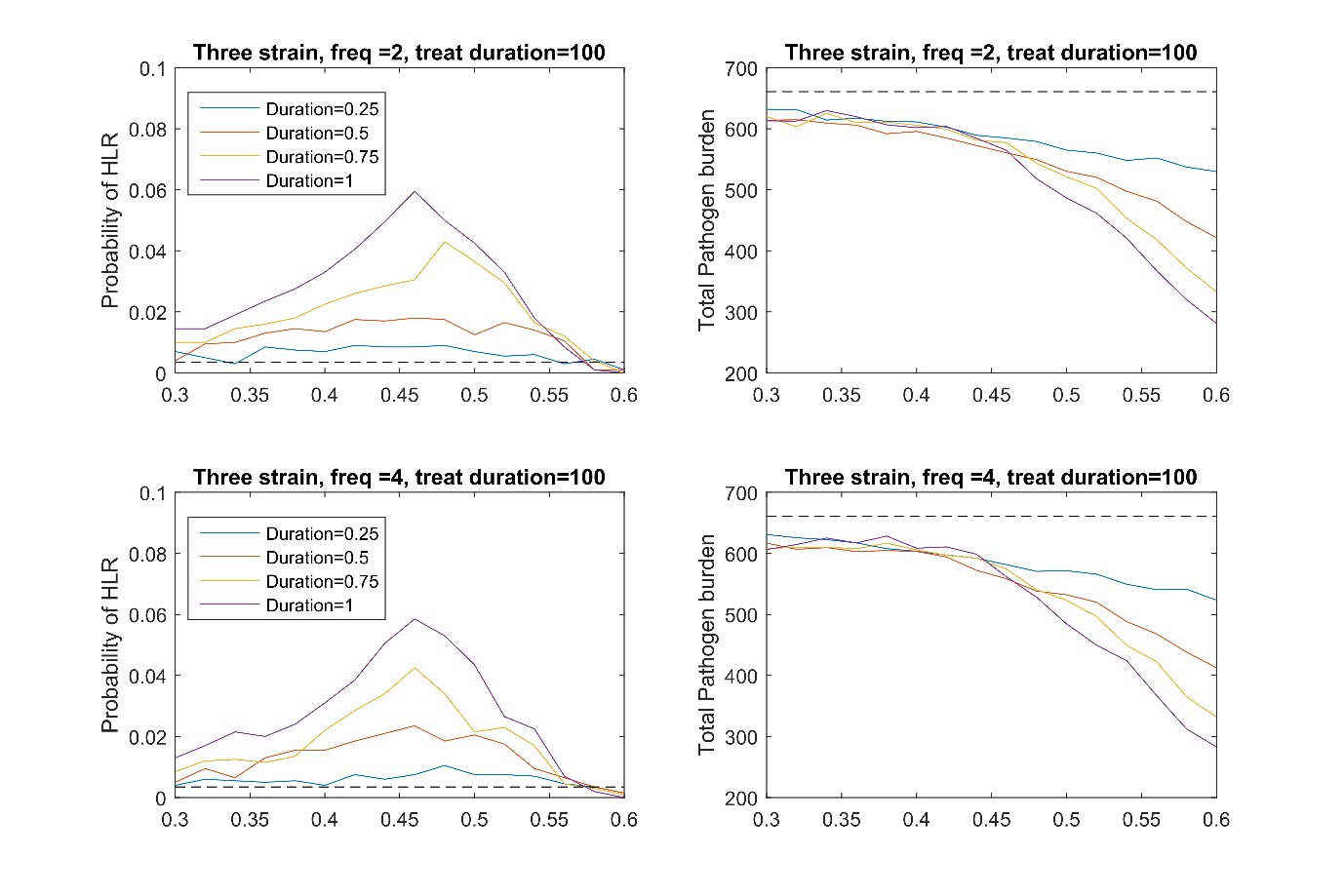
*

*Model 3: two strain with resource competition*


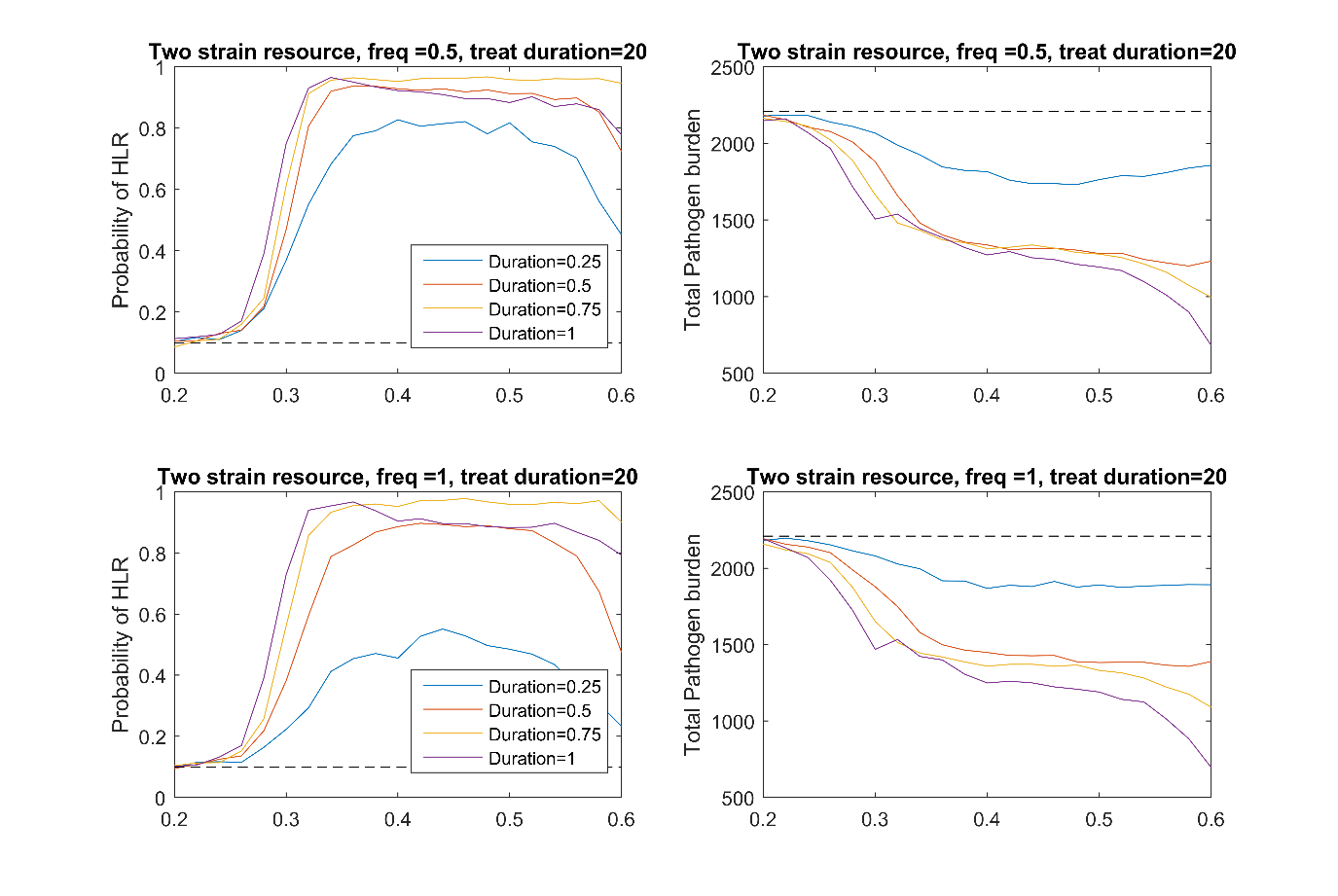

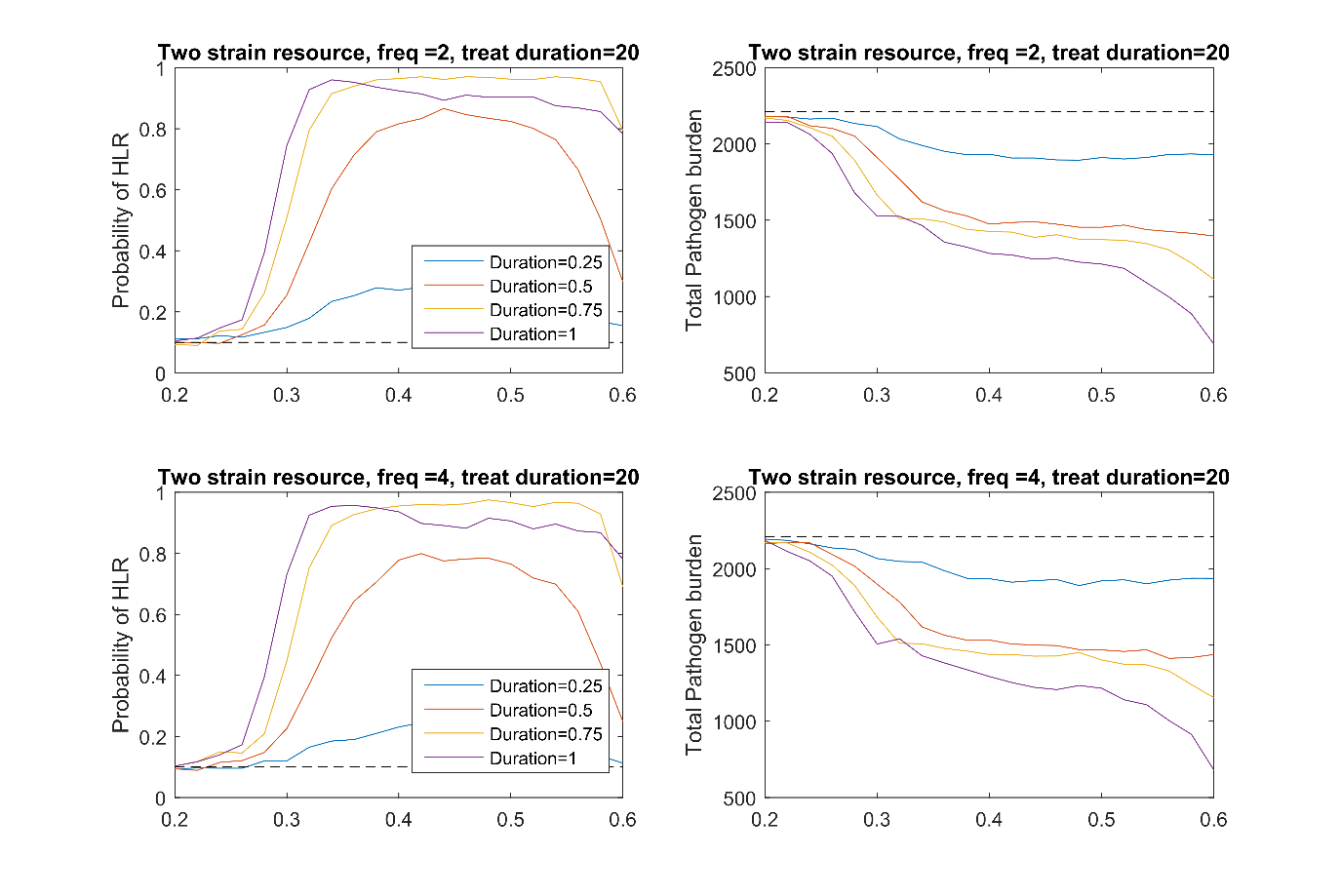


*Model 2: three strain with immune response and constant drug*

In these results we compare pulsed strategies with longer total treatment times to shorter, constant treatments. We do this by keeping the total ‘amount’ of drug constant. We use a pulse duration of 0.25 and we allow three pulses and we alter the pulse frequency. A frequency of 4 means that the pulses are consecutive, with a total treatment time of 0.75, while a frequency of 0.5 means the pulses are every second day, for a total of 6 days.

*
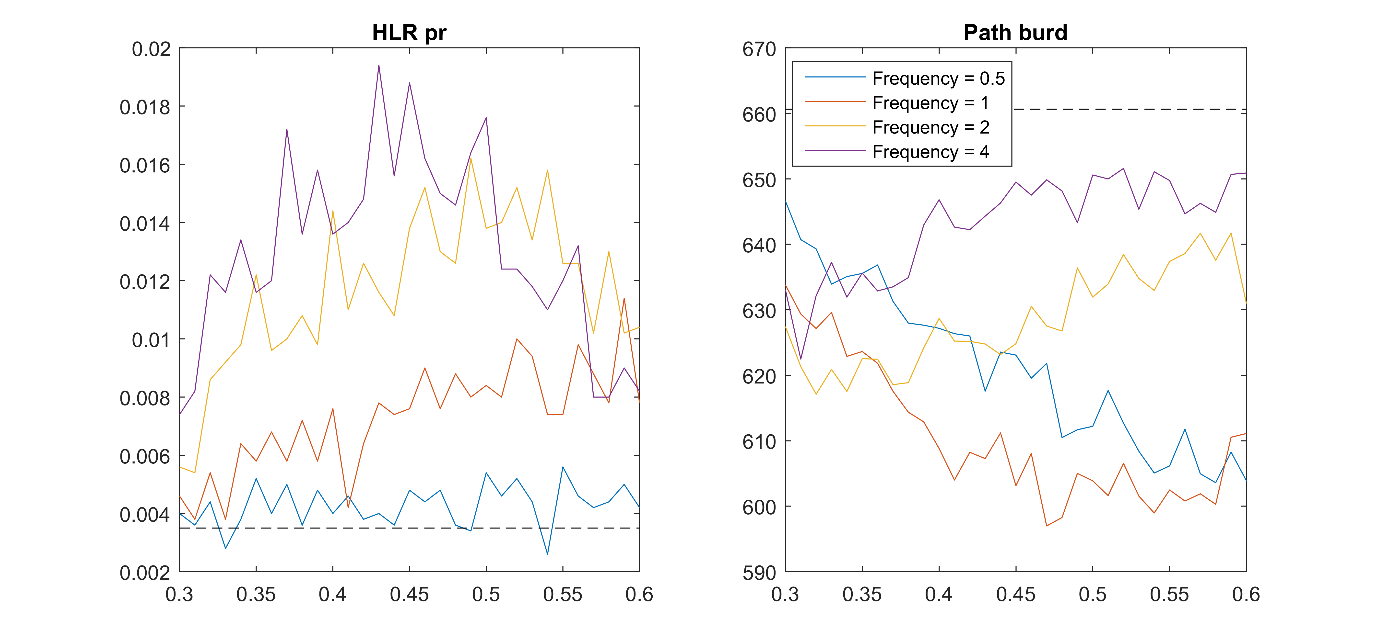
*
